# Characteristic Evaluation and Finite Element Analysis of a New Glass Fiber Post Based on Bio-Derived Polybenzoxazine

**DOI:** 10.3390/ijms26062444

**Published:** 2025-03-09

**Authors:** Phattarin Mora, Sarawut Rimdusit, Chanchira Jubsilp

**Affiliations:** 1 Department of Chemical Engineering, Faculty of Engineering, Srinakharinwirot University, Nakhonnayok 26120, Thailand; phattarin@g.swu.ac.th; 2 Research Unit in Polymeric Materials for Medical Practice Devices, Department of Chemical Engineering, Faculty of Engineering, Chulalongkorn University, Bangkok 10330, Thailand; sarawut.r@chula.ac.th

**Keywords:** dental composites, eco-friendly materials, phenolics, E-glass fibers, infrared spectroscopy, innovation

## Abstract

A new type of glass fiber (GF)-reinforced bio-derived polybenzoxazine (GF/bio-derived PBz) composites suitable for dental post applications was developed. The study assessed the effects of different quantities of GF on the mechanical and thermal characteristics, thermal stability, and flame resistance of the composite samples. Additionally, the feasibility of using GF/bio-derived PBz composites for dental posts was analyzed through finite element analysis (FEA). The stress distribution in a tooth model repaired with the newly developed GF/bio-derived PBz composite posts under oblique loads was compared to models repaired with conventional glass fiber post and gold alloy post. The incorporation of GFs significantly enhanced the flexural properties, thermal stability, and flame resistance of the composite samples, while also reducing thermal expansion in a manner that closely matched that of dentin. The FEA of a tooth model repaired with a composite post derived from GF/bio-based PBz revealed a stress distribution pattern comparable to that of a tooth model repaired using a conventional glass fiber post. Considering the composite’s mechanical properties, thermal stability, flame resistance, and its suitability for dental fiber posts as demonstrated by the FEA, the GF/bio-derived PBz holds significant promise for use in dental fiber post applications.

## 1. Introduction

Numerous advancements in material performance over the past decade can be credited to the evolution of multicomponent polymer systems, particularly through the use of multiphase materials like fiber and filler reinforcement in polymer matrix composites [1,2,3,4,5,6,7,8]. In recent times, thermosetting polymers have garnered significant interest from the automotive, aerospace, and construction sectors due to their substantial potential. Polybenzoxazines represent a novel category of phenolic polymers designed as an alternative to conventional high-performance thermosetting polymers, offering a diverse array of desirable attributes while addressing various limitations of traditional novolac and resole-type phenolic resins. They exhibit remarkable characteristics, including excellent stiffness, a relatively high glass transition temperature despite having a low cross-linking density, high char yield, and minimal water absorption. They can also be customized to achieve various properties, spanning from advanced epoxies and phenolic resins to bismaleimides and polyimides. In addition, their monomers, benzoxazines, demonstrate a low a-stage viscosity, facilitating formulation with a wide variety of fibers, minimal volumetric change during polymerization, they do not necessitate strong acid catalysts, and they do not generate any toxic by-products [9,10]. Due to the exceptional characteristics of polybenzoxazine, they are consistently employed as polymer matrices in fiber-reinforced composites that possess significant potential for use as heat-resistant, insulating, and structural materials, thanks to their outstanding thermal, mechanical, and electrical characteristics. Each polybenzoxazine/fiber composite exhibits unique properties that require appropriate processing techniques [11,12,13,14]. A thorough assessment has been conducted of the mechanical, thermal, and electrical attributes of these composites. Okhawilai et al. [15] examined the impact performance of the composite from glass and aramid fiber-reinforced polybenzoxazine/polyurethane composites. Their findings revealed that, with an equal number of plies, the deformation in the final panel of the S-glass composite was notably less than that of the E-glass composite specimen. These panels were evaluated for their resistance, and all specimens successfully withstood projectile penetration. The results obtained from this study demonstrate that these panels possess exceptional ballistic properties, making them suitable raw materials for body armor manufacturing. Advanced composite materials with mechanical integrity and self-extinguishing properties were presented by Jubsilp et al. [16]. They evaluated mechanical carbon fiber (CF) composites using polybenzoxazine modified with pyromellitic dianhydride as a matrix. The flexural modulus and strength of the composites were increased with an increase in CF content up to 65 wt% (57 vol%). The obtained flexural properties of the composite were still higher than those of carbon fiber-reinforced bisphenol-A-based epoxy composites. In addition, the carbon fiber composites also achieved the maximum V-0 fire-resistant classification. Wolter et al. [2] discovered that GF-reinforced polybenzoxazine composites exhibited commendable fire, smoke, and toxicity (FST) properties, comparable to or even superior to GF-reinforced epoxies in railway applications. Consequently, the developed composite has the potential to maintain elevated fire safety standards in industries such as railways, eliminating the need for additional flame-retardant additives. A fiber post composed of GF-reinforced petroleum-based polybenzoxazine was developed by Mora et al. [17,18]. The composites were found to be biocompatible materials as their cell viability was found to be more than 90%. Finite element analysis was also performed to investigate the complex mechanical reactions of the glass fiber-reinforced polybenzoxazine composites under external loads. This approach is recognized for its modern technological benefits, facilitating the modeling process to be straightforward, convenient, and fast. However, the escalating global fossil fuel crisis, along with ongoing health and environmental concerns, presents significant challenges for polybenzoxazines regarding their availability and pricing. Additionally, petroleum does not readily provide a diverse range of chemical structures or functional properties. Recent research initiatives have concentrated on creating renewable substitutes to replace the phenol and amine monomers found in benzoxazines. Bio-based phenols, including eugenol [19,20,21], vanillin [22,23,24,25,26], and cardanol [27,28,29,30], have been utilized as raw materials in the production of bio-based polybenzoxazines, whereas for bio-based amines, furfurylamine and stearylamine are the preferred choices [31,32,33]. The aim is to create a green process that transforms these raw materials into final products, with the goal of developing a bio-based and biodegradable polybenzoxazine that aligns with the principles of green chemistry [10].

In recent years, research has focused on the use of bio-based polybenzoxazine as a matrix for fiber-reinforced composites, exploring its potential applications in various fields such as coatings, construction, and the transportation industry, including automotive and aerospace interior panels. Using glass fiber-reinforced eugenol/furfurylamine-derived benzoxazine/epoxidized castor oil (E-fa/ECO) copolymers activated by near-infrared light, a new sustainable self-healing polymer composite was created [3]. The thermal properties and stability of the sustainable composites showed significant improvements with the addition of reinforcing glass fiber, and the flexural strength of the composites was also enhanced by incorporating GF, reaching levels of up to 70 wt%. In addition, the composites demonstrated effective macroscopic thermal healing capabilities, achieving results between 64% and 86%, suggesting their possible application as building lath. Luengrojanakul et al. [22] studied the effects of adding CF and graphene nanoparticles (GnPs) on the mechanical and shape memory properties of vanillin/furfurylamine-based polybenzoxazine (V-fa)/ECO composite. The results demonstrated that adding GnPs and CF enhanced both the flexural strength and modulus due to improved interfacial adhesion and fiber reinforcement, with the best results seen at 3 wt% GnPs and 60 wt% CF. Furthermore, the optimal compositions for achieving the best performance of the bio-based SMP composite were found to be 40 wt% CF and 3 wt% GnPs. The eugenol/furfurylamine-derived polybenzoxazine (E-fa) and eugenol/stearylamine-derived polybenzoxazine (E-sa) and jute fiber (a bio-fiber) were used to prepare bio-based polybenzoxazine/jute fiber composites [21]. It was found that the modulus and glass transition temperature of the E-fa and E-sa were enhanced with the addition of jute fiber. Previous research indicates that reinforcing bio-based polybenzoxazine with glass fiber (GF) enhances its mechanical and thermal properties sufficiently to meet the necessary characteristics for fiber post materials. This includes physical properties like the modulus of elasticity, flexural strength, and thermal expansion, which closely resemble those of dentin. Furthermore, the use of bio-based polybenzoxazine as a substitute for epoxy resins—commonly used as the polymer matrix for glass fiber posts—offers a solution to the drawbacks associated with epoxy resins, such as the use of toxic hardeners and potential irritation to the eyes and skin from prolonged exposure. In addition, it is anticipated that the benefits of glass fiber-reinforced bio-based polybenzoxazine posts will be realized in several areas, including their ease of handling, mechanical properties, esthetic appeal, removability, and their potential as biocompatible materials.

Therefore, in this work, a new polymer composite post has been developed utilizing a vanillin/furfurylamine-based polybenzoxazine, reinforced with GF. The effect of GF content on the mechanical and thermal characteristics, thermal stability, and flame resistance was evaluated. In addition, the mechanical response to externally applied loads of the composite post, utilizing FEA, was assessed. The findings from the simulations will be contrasted against data from commercially available GF post and gold alloy post.

## 2. Results and Discussion

### 2.1. Characteristics of V-Fa and Poly(V-Fa)

**Figure 1:**
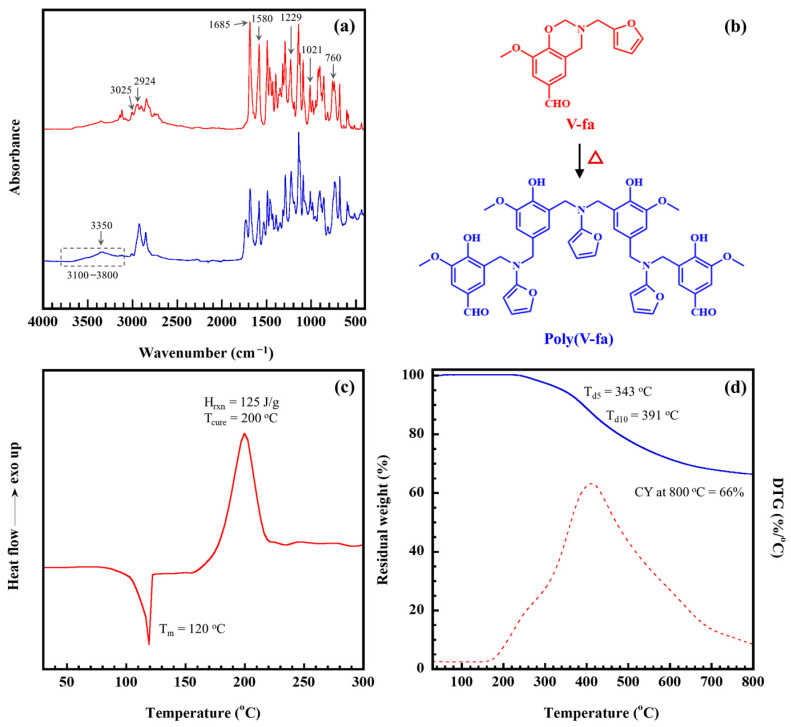
Characteristics of V-fa and poly(V-fa): (**a**) FT-IR spectrum of V-fa, (**b**) Structure of the synthesized V-fa, (**c**) DSC thermogram of V-fa, (**d**) TGA thermogram of poly(V-fa).

FT-IR spectroscopy was used to examine the structure of the synthesized V-fa. The V-fa spectrum is shown in Figure 1a. Distinct absorption peaks associated with furan rings are identified at 1580 and 760 cm^−1^. The peak observed at 1685 cm^−1^ confirms the existence of the formyl group in vanillin. Additionally, the absorption peak at 1021 cm^−1^, which corresponds to the asymmetric stretching of C-N-C, and at 1229 cm^−1^, indicative of the Ar-C-O stretch, suggests that an oxazine ring is integrated with vanillin’s benzene ring [23,34]. In addition, the weak bands observed at 3025 and 2924 cm^−1^ are attributed to the asymmetric and symmetric stretching of the aromatic -CH group, respectively. These findings thereby validate the formation of V-fa, as shown in Figure 1b.

The curing characteristics of V-fa were examined using differential scanning calorimetry (DSC). The DSC thermogram for V-fa is depicted in Figure 1c. It was found that the endothermic peak at 120 °C indicates the melting temperature (T_m_) of V-fa, while the exothermic trend sheds light on the curing process. The curing begins at an onset temperature (T_onset_) of 160 °C, reaches its maximum curing temperature (T_max_) at 200 °C, and completes at the final curing temperature (T_final_) of 230 °C. This indicates that achieving a temperature of 250 °C is essential for the full polymerization of V-fa. Additionally, the total heat released during the curing/polymerization process is calculated to be 125 J/g. Thus, the curing behavior of V-fa occurs at a temperature that is 30 °C lower than the conventional petroleum-based benzoxazine, specifically the bisphenol-A/aniline-based benzoxazine (BA-a), which has a T_max_ of 230 °C [35]. This reduction in curing temperature suggests that the formyl group present in vanillin plays a significant role, as it has been previously noted that it can easily oxidize to a carboxylic group, which facilitates the ring-opening polymerization of V-fa [36,37]. Following the curing process, polymerized V-fa was obtained, as evidenced in Figure 1a, which displays a broad peak around 3100–3800 cm^−1^ and a distinctive band at 3350 cm^−1^ corresponding to the hydroxyl group produced by the thermal ring-opening reaction of the oxazine ring. The proposed curing reaction of the V-fa is also exhibited in Figure 1b.

The thermal stability of poly(V-fa) was evaluated through thermogravimetric analysis (TGA), as shown in Figure 1d. This figure illustrates the weight loss of poly(V-fa) in correlation with temperature and includes its derivative thermogravimetric curve (DTG). The thermogram indicates that degradation occurs in two steps, with poly(V-fa) maintaining thermal stability up to 230 °C. The first step of the degradation of poly(V-fa) transpires between 200 and 300, while the second step transpires between 300 and 700 °C. The temperature at which 5% degradation (T_d5_) is observed is 343 °C, while 10% degradation (T_d10_) is detected at 391 °C. These observations suggest that the degradation temperature of poly(V-fa) closely resembles that of bisphenol-A/aniline PBz, i.e., T_d5_~337 °C [38]. Additionally, a char yield of 66% was achieved after heating to 800 °C, demonstrating a higher char yield compared to conventional dibenzene-based polybenzoxazines, such as those derived from bisphenol-A, which typically show a char yield between 28% and 32% at 800 °C [9]. This suggests that the network incorporating furan significantly enhances char formation in polybenzoxazines.

### 2.2. Flexural Properties of GF/Poly (V-Fa) Composites

**Figure 2:**
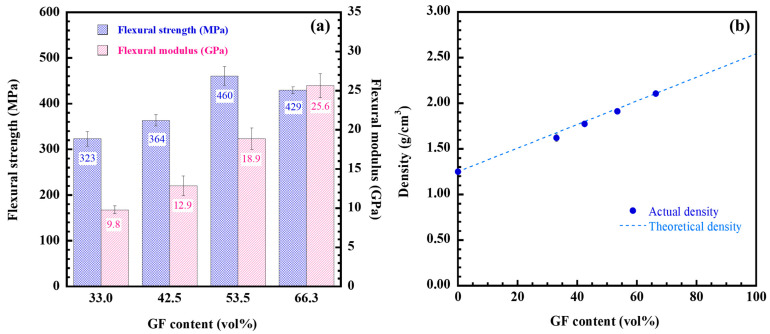
(**a**) Flexural property of poly(V-fa) composites at various GF contents, (**b**) Density of GF-reinforced poly(V-fa) composites at various GF contents: (●) Actual density, (---) Theoretical density.

As seen in Figure 2a, the effect of GF content on the flexural characteristics of poly(V-fa) composites was investigated. As more glass fiber was added—between 33.0 and 42.5 vol%—the strength of the composites rose significantly, from 323.2 ± 16.2 to 460.4 ± 20.4 MPa, respectively. This increase in flexural strength can be attributed to a robust interfacial bond between the glass fiber and the polymer matrix [17,18,34]. However, with an increase to 66.3 vol% glass fiber content, the strength slightly declined to 429.5 ± 7.7 MPa. The slight decline in strength is probably attributed to the reduced content of the poly(V-fa) matrix. This reduction affects the GF/poly(V-fa) interface, which is a crucial factor impacting on the overall macroscopic properties of the composite, including its strength, as it leads to diminished stress transfer between the GF and poly(V-fa). The results suggested that the reinforcement of GF at 53.5 vol% into poly(V-fa) showed high flexural strength for further dental post material applications. Furthermore, the flexural modulus of the composites was found to improve with higher GF content, exhibiting values that ranged from 9.8 ± 0.5 to 25.6 ± 1.52 GPa. The necessary elastic modulus, which is a crucial factor for load transmission, should therefore be close to that of dentin, i.e., 18 to 40 GPa [39,40,41,42], in order to use the glass fiber-reinforced poly (V-fa) composite as a glass fiber post. Since the experimental density of the GF/poly(V-fa) composites followed the rule of mixture, as shown in Figure 2b, the enhancement of the composites’ flexural properties with rising GF content aligned with their experimental density.

### 2.3. Thermal Expansion of GF/Poly(V-Fa) Composites

**Figure 3:**
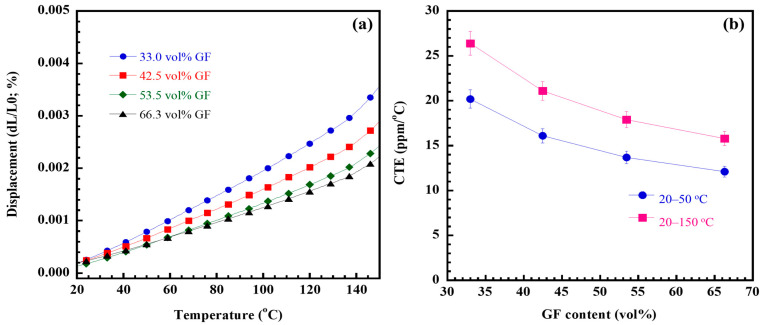
(**a**) TMA curves of GF/poly(V-fa) composites at various GF contents; (**b**) CTE of the composites in the glassy region from 20 to 50 °C and from 20 to 150 °C.

The coefficient of thermal expansion (CTE) of restorative materials for dental restoration was analyzed due to their expansion upon heating from hot foods and beverages, along with contraction when exposed to cold. This disparity in CTE between the dental tissue and the restorative materials can lead to fractures during the cycles of expansion and contraction. To explore their potential use as new dental fiber posts, the CTE values of GF/poly(V-fa) composites were evaluated. Figure 3a depicts the displacement of the composite samples ranging from 33.0 to 66.3 vol% GF over a temperature range of 20 to 150 °C. All of the tested composites exhibited greater displacement with increasing temperature; however, at a specific temperature, higher GF loadings resulted in reduced displacement of the samples.

Figure 3b presents the average CTE values for the glassy state of the GF/poly(V-fa) composite samples, as derived from the slopes in Figure 3a for the temperature ranges of 20 to 50 °C and 20 to 150 °C. The CTE for the composite samples over the 20–150 °C interval was observed to reduce their CTE values with an increase in GF content. Specifically, the CTE values dropped to 26.4, 21.1, 17.9, and 15.8 ppm/°C for composites containing 33.0, 42.5, 53.5, and 66.3 vol% GF, respectively. Likewise, during the 20–50 °C range, the CTE of the composite samples also showed a decline with rising GF content from 33.0 vol% to 66.3 vol%, recording values of 20.2, 16.1, 13.7, and 12.1 ppm/°C, respectively. The decreased CTE values of the composite samples can be attributed to the inherently lower CTE value of GF compared to the polymer matrix, specifically ranging from 5 to 12 ppm/°C. Additionally, the higher rigidity of the GF, with a modulus of 70 to 85 GPa, contributes to the limited motion of the macromolecular segments in poly(V-fa). Furthermore, the interfacial adhesion between the GF and the poly(V-fa) matrix significantly influences the reduction in the CTE of the composites. In comparison with GF/poly(BA-a) composites [17], the GF/poly(V-fa) composites showed high performance with lower CTE values than that of GF/poly(BA-a) composites, which would cause lower thermal stress and high dimensional stability.

In order to utilize GF/poly(V-fa) composites as dental glass fiber posts in tooth restoration, it is crucial to compare their CTE values with those of dental tissues, like dentin, rather than evaluating the CTE of each material individually. The analysis indicated that the CTE of the composite containing 33.0 vol% and 66.3 vol% was approaching that of dentin, which is approximately 11 ppm/°C at temperatures between 10 and 80 °C [43], typical for tooth exposure during the consumption of hot beverages and food. As a result, the CTE for poly(V-fa) composite with the 53.5 vol% and 66.3 vol% closely matches that of dentin, which helps minimize stress buildup at the dentin–post interface and effectively prevents the loss of marginal adaptation.

### 2.4. Thermomechanical Properties of GF/Poly(V-Fa) Composites

Using dynamic mechanical analysis (DMA), the viscoelastic characteristics of the GF/poly(V-fa) composites with different GF contents were investigated. Table 1 lists key DMA metrics for the composite samples, including the temperature-dependent damping factor (tan δ), loss modulus (E″), and storage modulus (E′). The E′ values in the glassy state of the composite samples, known as the dynamic modulus, indicate the material’s stiffness and elastic behavior, and they showed an upward trend with higher GF contents. These findings align well with the flexural properties discussed earlier. On the other hand, the glass transition temperature (T_g_) is determined as the localized maximum in the loss modulus, which is a crucial factor concerning molecular mobility. As the composite samples approach and surpass the peak, the dissipated energy rises due to the relatively extensive segmental movement of the poly(V-fa).

From Table 1, it can be seen that the amount of GF in the GF/poly(V-fa) composite samples increased, and the T_g_ of the composite samples also rose. Specifically, the T_g_ values for the composite samples, deduced from E″, were noted to range from 164 to 170 °C. This increase in T_g_ can be linked to the GF’s capacity to enhance interfacial adhesion within the composite, which in turn supports the movement of poly(V-fa) molecular chains, much like what has been studied in GF/poly(BA-a) composites [18] and GF/poly(E-fa) composites [44]. The working temperature of dental materials in contact with human teeth exposed to hot foods and drinks was found to be around 36 °C to 48 °C [45], and the actual intraoral temperature varies between approximately 5 °C and 55 °C [43]. Consequently, it was noted that the glass transition temperature (T_g_) of the GF/poly(V-fa) composite samples exceeded the temperature range of 5–55 °C, suggesting that the composites exhibit strong mechanical properties at working temperatures.

### 2.5. Thermal Stability of GF/Poly(V-Fa) Composites

**Figure 4:**
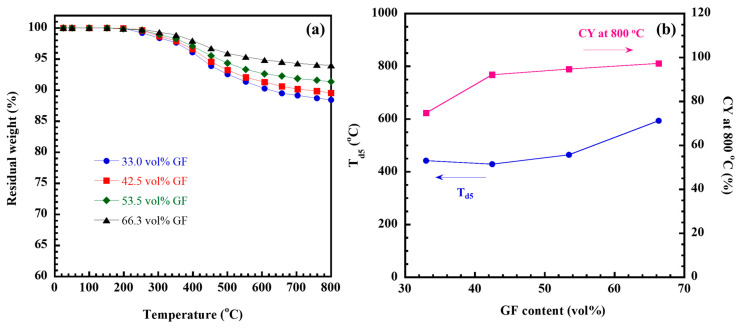
(**a**) TGA thermograms and (**b**) T_d5_ and CY at 800 °C of GF/poly(V-fa) composites at various GF contents (vol%).

The thermal degradation of fiber-reinforced polymer composites plays a vital role in structural applications by offering valuable information about material performance, load-bearing capacity at specific temperatures, and dimensional stability when exposed to high temperatures. As a result, assessing the thermal stability of restorative materials is essential for determining their capability to endure the elevated temperatures generated during tooth restoration without undergoing decomposition. To evaluate the thermal stability of the GF/poly(V-fa) composites, the TGA thermogram of poly(V-fa) composites containing varying amounts of GF is shown in Figure 4a, while the data on thermal degradation at 5% weight loss (T_d5_) and residual weight or char yield (CY) at 800 °C for the poly(V-fa) composites are illustrated in Figure 4b.

It was noted that as the GF content in the poly(V-fa) composites increased, the T_d5_ also rose. This indicates that the incorporation of GF enhances the thermal stability of the poly(V-fa) composites, as the degradation temperature of GF exceeds 800 °C [46]. This reinforcement prevents the direct thermal degradation of poly(V-fa) by providing a shielding effect against heat. Additionally, GF plays a role in impeding the release of volatile organic compounds during the thermal breakdown of poly(V-fa). When evaluating the working temperature of dental materials in relation to human teeth exposed to hot foods and beverages (approximately 36 to 48 °C), as well as the maximum oral temperatures recorded around the front teeth due to hot liquids (around 70 °C), it was found that the degradation temperature of poly(V-fa/ECO) composites across all ratios of GF reinforcement was higher.

Furthermore, the CY of the poly(V-fa) composites at 800 °C in nitrogen atmosphere, as depicted in Figure 4b, also showed an increase from 74.7% to 97.3% with the addition of GF from 33.0 vol% to 66.3 vol%. This property arises from the mineral composition of GF, which is inherently non-combustible and does not release any toxic substances when subjected to heat. Consequently, the high char yield obtained indicates that the GF/poly(V-fa) exhibits outstanding thermal stability and is likely to demonstrate effective flame retardancy.

To assess the flame-retardant properties of the GF/poly(V-fa) composites, the limiting oxygen index (*LOI*) serves as a key measurement. It can be determined by using the *CY* obtained during pyrolysis in nitrogen atmosphere combined with the Van Krevelen and Hoftyzer equation (Equation (1)).

(1) LOI=17.5+0.4CY

As a result, the poly(V-fa) composites reinforced with glass fiber exhibit a char yield ranging from 74.7 to 97.3, resulting in LOI values between 47.4 and 56.4. These parameters indicate that the composites demonstrate self-extinguishing characteristics.

### 2.6. Feasibility of GF/Poly(V-Fa) Composites for Dental Fiber Post by FEA

**Figure 5:**
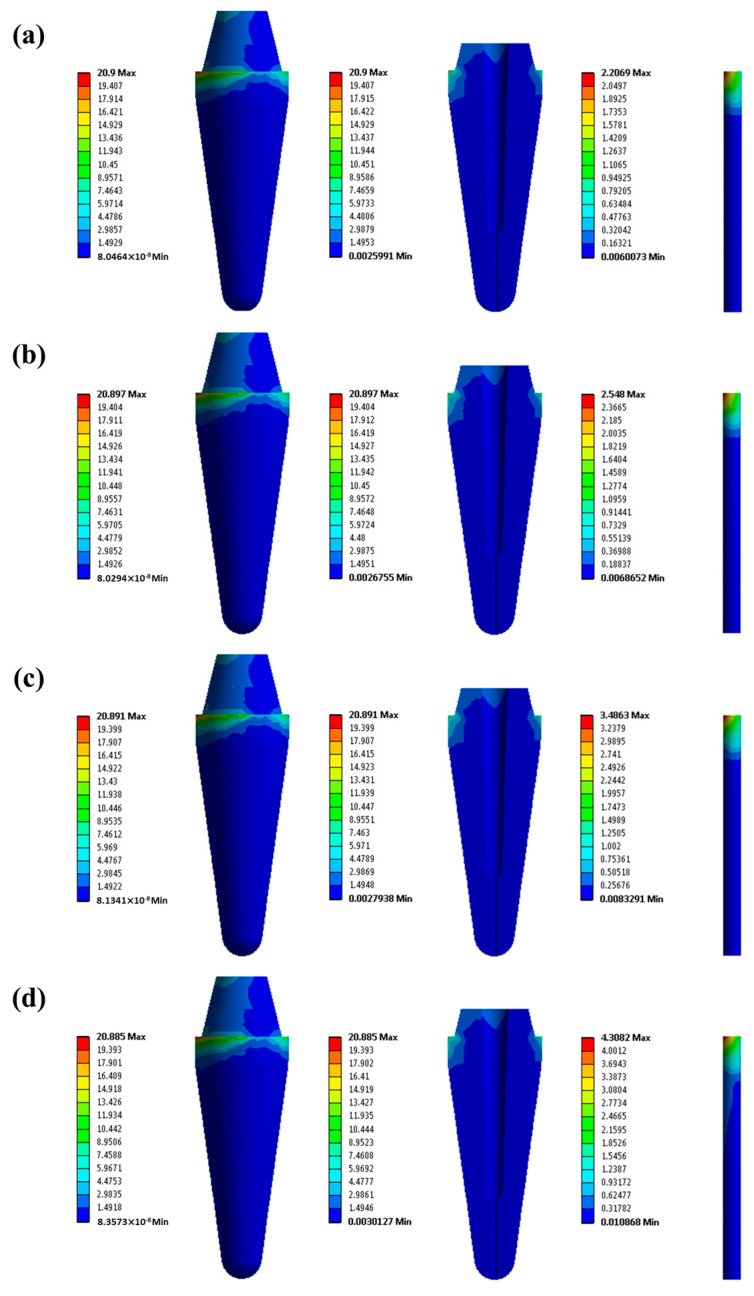
σ_v_ distribution of tooth restored with GF/poly(V-fa) composite post at various GF contents: (**a**) 33.0 vol%, (**b**) 42.5 vol%, (**c**) 53.5 vol%, and (**d**) 66.3 vol%.

Figure 5 shows the von Mises stress (σ_v_) distribution in the root dentin structure and the post for the GF/poly(V-fa) composite posts with various GF contents. The magnitude of the maximum σ_v_ in the tooth structures and in the post was also recorded. It can be seen that the maximum σ_v_ presented at the dentin region and cervical thirds of the root and reduced from 20.900 to 20.885 MPa for the composite samples reinforced with GF of 33.0–66.3 vol%, respectively. The reduction in maximum σ_v_ in the dentin region caused by the composite post reinforced with a higher GF contents could be carried by the larger load fraction, i.e., 2.2069, 2.548, 3.4863, and 4.3082 MPa. This behavior was similar to the tooth restored with GF post based on bisphenol-A/aniline polybenzoxazine [18]. GF/poly(V-fa) composite at 53.5 vol% GF may be the material for a post application given its dentin-like elastic modulus and lower stress generation in the remaining dentin.

**Figure 6:**
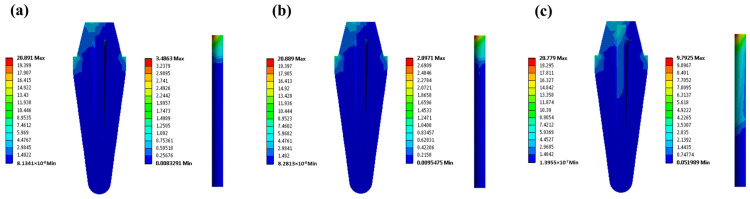
σ_v_ distribution of tooth restored with posts: (**a**) 53.5 vol% GF/poly(V-fa) composite post, (**b**) commercial GF post, and (**c**) gold alloy post.

To evaluate the performance of the developed 53.5 vol% GF/poly(V-fa) composite post in comparison to commercial GF and gold alloy posts, the σ_v_ distribution on a restored tooth model was assessed and is presented in Figure 6. It was observed that all the models showed the highest σ_v_ in the cervical region of the dentin. Notably, the model restored with the composite post displayed a maximum σ_v_ value of 28.267 MPa, which was similar to 28.320 MPa for the model using the commercial GF post. The model restored with gold alloy post showed the lower maximum σ_v_, i.e., 20.799 MPa, than the model restored with GF posts. The results indicate that the GF/poly(Vf-a) composite post can support a greater load fraction due to its elastic modulus being comparable to that of dentin. Furthermore, compared to restorations using gold alloy posts, the minimal color change seen at the apical third of the root indicates effective occlusal load dissipation, greatly lowering the risk of vertical fractures.

## 3. Materials and Methods

### 3.1. Materials

Vanillin (99%) and furfurylamine (>99%) were sourced from Sigma-Aldrich Pte. Ltd. in Singapore, while paraformaldehyde (AR grade) was obtained from Merck Co., Ltd. based in Darmstadt, Germany. All chemicals were utilized in their received state. Additionally, plain weave glass fiber (GF) fabrics with an areal density of 600 g/m^2^ were acquired from Thai Polyadd Ltd. Partnership (Bangkok, Thailand).

### 3.2. Benzoxazine Resin, Prepreg, and Composite Preparation

**Figure 7:**
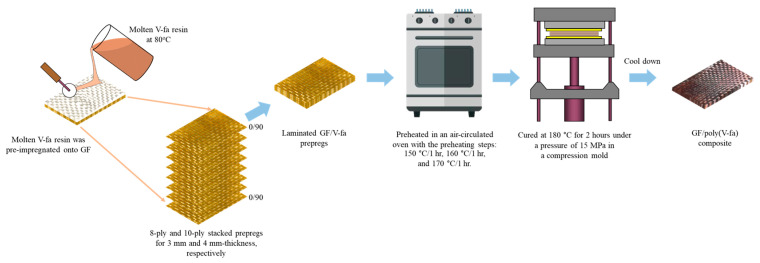
Preparation of GF/poly(V-fa) composites.

The bio-derived benzoxazine resin (V-fa) was synthesized through a solvent-free process utilizing biobased raw materials, vanillin, furfurylamine, and paraformaldehyde, in a molar ratio of 1:2:1. The reaction generated a dark brown liquid resin, achieved by continuously mixing the three ingredients for 40 min at 110 °C. The GF/poly(V-fa) composites using different GF contents of vol% were prepared as illustrated in Figure 7. The molten V-fa resin was then pre-impregnated onto GF at a temperature of 80 °C. Subsequently, the composite laminates underwent a preheating process at temperatures ranging from 150 to 170 °C for 3 h, before being cured at 180 °C for 2 h under a pressure of 15 MPa in a compression mold. The cured V-fa was characterized after cooling down to room temperature. The differential scanning calorimetry (DSC) technique indicated a complete curing conversion of 100% for the GF/poly(V-fa) composites, demonstrating that the composites were fully cured.

The GF volume percentage in the poly(V-fa) composites $\left(V_{f}\right)$ can be calculated through Equation (2):

(2) $$V_{f}=\frac{W_{f}/\rho_{f}}{W_{f}/\rho_{f}+W_{m}/\rho_{m}}$$

where $W_{f}$ is the weight of GF, $W_{m}$ is the weight of poly(V-fa), $\rho_{f}$ is the density of GF, and $\rho_{m}$ is the density of poly(V-fa). The $\rho_{f}$ and $\rho_{m}$ are 2.54 and 1.25 g/cm^3^, respectively.

### 3.3. Samples Characterization 

The composite density is assessed through the water displacement method, following the guidelines set by ASTM D792-20 [47]. The three samples were tested, and their average density values were reported.

A differential scanning calorimeter, DSC1 Module from Mettler Toledo Ltd. (Bangkok, Thailand), was employed to measure the melting temperature (T_m_) and curing characteristics of V-fa. Using a sample weight of 5 mg contained in an aluminum pan, the experiments were carried out in a N_2_ environment at a heating rate of 10 °C/min.

The Universal Testing Machine was used to analyze the composite’s flexural properties in compliance with ASTM D790M-93 [48]. The specimens’ measurements were 60 mm × 25 mm × 3 mm. A three-point bending test with a 48 mm support span and a crosshead speed of 1.2 mm/min was performed. The support span-to-thickness of the sample’s ratio is 16:1. Five samples of each composite composition were tested, and the average values were reported.

The dynamic viscoelastic analyzer (model DMA1, Mettler Toledo) was used to assess the samples’ dynamic mechanical characteristics. Throughout the process, a three-point bending test was used. At a frequency of 1 Hz and an amplitude of 30 μm, strain was measured. The samples, which had dimensions of 10 mm × 50 mm × 3 mm, were heated from 30 to 300 °C at a rate of 2 °C/min.

The Mettler Toledo thermogravimetric analyzer (model TGA1 Module) was used to evaluate the samples’ thermal stability. During the analysis, the specimens were heated from 25 to 800 °C at a rate of 20 °C/min while being continuously supplied with 50 mL/min of N_2_.

A thermomechanical analyzer (TMA) (Bruker-AXS, TMA 4010, Bangkok, Thailand) was employed to determine the CTE of the samples. The specimens, measuring 10 mm × 5 mm × 4 mm, were examined at an average heating rate of 5 °C/min within the temperature range of 100–200 °C, applying a constant load of 5 mN (0.5 g) in a nitrogen environment. The CTE for each sample was obtained by averaging three measurements.

### 3.4. Finite Element Analysis (FEA)

**Figure 8:**
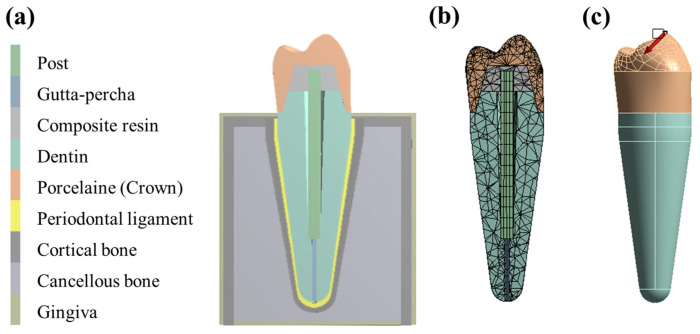
(**a**) Tooth model repaired with GF/poly(V-fa) composite, commercial GF, and gold alloy posts, (**b**) 3D mesh of tooth model, and (**c**) 100 N oblique load at 45° angle.

ANSYS Inc., in Canonsburg, Pennsylvania-based company, used the Design Modeler feature in ANSYS Workbench 2022 R1 (file version 22.1.0.2021111419) software to create the 3D model. The GF/poly(V-fa) composite post, gutta-percha, composite resin, dentin, crown, periodontal ligament, cortical bone, cancellous bone, and gingiva are all included in the model of the endodontically treated tooth, as shown in Figure 8a. The analysis of the 3D finite element method (FEM) was conducted using ANSYS Workbench 2022 R1 software. Subsequently, a 3D mesh was developed consisting of solid elements characterized by nodes. For the tooth restored with post models, there were 7074 elements and 13,195 nodes created (Figure 8b). A 100 N axial load was applied obliquely from the buccal to the lingual region at a 45° angle to the tip of the buccal cusp (Figure 8c). The outer surface of the bone could not move freely in any of the models.

Table 2 lists all of the materials and structures that were categorized as isotropic, homogeneous, and linearly elastic. These included gutta-percha, composite resin, dentin, crown, periodontal ligament, cortical bone, cancellous bone, gingiva, and gold alloy. On the other hand, GF/poly(V-fa) composites, which are used as glass fiber posts, are classified as orthotropic materials, which means that their properties vary along three orthogonal axes, as Table 3 illustrates.

## 4. Conclusions

GF-reinforced composites made from bio-derived polybenzoxazine, which is synthesized from vanillin and furfurylamine, were successfully developed. The addition of GF positively affected the mechanical and thermal properties, with notable improvements in flexural modulus and strength, glass transition temperature, degradation temperature, and flame resistance observed as the GF content increased. Furthermore, GF reinforcement significantly reduced the CTE due to the robust structural integrity of GF within the bio-derived polybenzoxazine matrix. All composite samples exhibited LOI values significantly surpassing the threshold for self-extinguishing materials. The GF-reinforced bio-derived polybenzoxazine post exhibits an elastic modulus that aligns more closely with that of dentin, in contrast to the significantly higher modulus of a conventional glass fiber post and gold alloy post. Therefore, this study suggests that GF-reinforced bio-derived polybenzoxazine could be an excellent choice for post applications that demand both strong mechanical and thermal properties, and environmentally friendly materials.

## Figures and Tables

**Table 1:** Thermomechanical properties of GF/poly(V-fa) composites.

Composite Samples	E′ (GPa)	T_g_ from E″
33.0 vol% GF/poly(V-fa)	12.5	164
42.5 vol% GF/poly(V-fa)	15.4	167
53.5 vol% GF/poly(V-fa)	18.0	170
66.3 vol% GF/poly(V-fa)	19.8	170

**Table 2:** Elastic characteristics of the isotropic materials input into this study [17,37,49].

Material	Elastic Modulus	Poisson’s Coefficient
Gutta-percha	6.9 × 10^−4^	0.45
Composite resin	16.6	0.24
Dentin	18.6	0.31
Porcelain (crown)	120	0.28
Periodontal ligament	6.89 × 10^−2^	0.45
Cortical bone	13.7	0.30
Cancellous bone	1.37	0.30
Gingiva	19.6 × 10^−3^	0.30
Gold alloy	93.0	0.33

**Table 3:** Elastic characteristics of the orthotropic materials input into this study.

Elastic Constant	GF/Poly(V-Fa) Composite Post at Various GF Contents (vol% (wt%))				Commercial GF Post [49]
	33.0 (50)	42.5 (60)	53.5 (70)	66.3 (80)	
E_L_, GPa	9.80	12.90	18.90	25.60	37.0
E_T_ = E_T′_, GPa	6.39	7.92	10.31	14.59	9.50
G_LT_ = G_LT′_, GPa	3.58	4.32	5.47	7.50	3.10
G_TT′_, GPa	2.44	3.05	4.03	5.82	3.50
ν_LT_ = ν_LT′_	0.25	0.24	0.23	0.22	0.27
ν_TL_ = ν_T′L_	0.17	0.15	0.13	0.12	0.27
ν_TT′_	0.31	0.30	0.28	0.25	0.34

Note: E_L_ obtained from experimental data, as reported in item 2.2, while the other elastic properties were calculated theoretically using the equations presented by Pegoretti et al. [49]. The elastic moduli for poly(V-fa) and glass fiber are 3.09 GPa and 72 GPa, respectively.

## Data Availability

Data are contained within the article.
